# Paper on Cancrum Oris

**Published:** 1872-04

**Authors:** C. S. Kittredge

**Affiliations:** Oakland


*Paper on Cancrum Oris.* By C. S. Kittredge, M.D., of Oakland. Read before the Alameda County Medical Association, March 4th, 1872.

During the years 1863–64, it was my privilege to be the assistant of the late and much lamented Henry Whittlesey, M.D., then the resident physician at the Nursery Hospital on Randall's Island, New York City.

It was our fortune to have during the year, a large number of cases of cancrum oris, and our conclusions with reference to this peculiarly fatal disease may be of interest to the profession.

This formidable affection—which is a malignant form of ulcerative stomatitis—is a disease of debility, induced in children under five years of age, predisposed to tuberculosis by a want of cleanliness, proper food, and care; and appears chiefly in those who are received into our hospitals from the alms-houses, jails and poorly ventilated lodgings—those dark, damp, filthy apartments, where congregate the vice and destitution of our large cities.

It appears generally as the sequel to rubeola, scarlatina, or some other debilitating disease. It occurs at all seasons of the year, but is most prevalent during the summer months. That it is a contagious disease, though in a limited degree, cannot be doubted, yet I believe that only those who are badly nourished, and of a tuberculous or scrofulous diathesis, are liable to be affected.

*Mode of access.* The system being in a morbid state, a simple shallow ulcer makes its appearance on the inside of the cheek, or between the two incisor teeth of the upper jaw, at the junction of the frænum labii superioris with the gums, causing the lips to swell rapidly and enormously, the teeth to loosen and drop out, and a general destruction of the parts; or it may commence in the mucous membrane of the throat, or roof of the mouth, destroying everything in its course.

A description of this disease as it appears and progresses when commencing in the cheek—its most common form—will suffice to show all its characteristic features. The first thing noticeable, is a slight circumscribed swelling, hard, painless, red, tense and shining, resembling somewhat the sting of a bee. A very small whitish ulcer will now be found on the inside of the cheek, corresponding in position with the centre of the external induration. The breath is slightly fœtid, and an increase in the secretion of saliva is apparent. The ulcer very soon becomes ash-colored, and rapidly spreads, assuming a phagedenic character, discharging an offensive dirty-colored fluid. In two or three days, a black depressed spot appears on the outside of the cheek, corresponding in size and position with the ulcer within. In the course of another day, a small hole is seen in the centre of this gangrenous spot, through which oozes, drop by drop, the offensive acrid discharge. The face now assumes a peculiar ashy-yellow color, swells greatly, frequently to such an extent as to close both eyes; mortification goes on rapidly, often involving the whole cheek, and exposing the cavity of the mouth. The bones of the face may become affected with necrosis, the teeth fall out, and the little patient becomes a most frightful spectacle to behold, while the horrible stench that arises is well nigh unbearable. The constitutional symptoms are extreme prostration, with rapid, feeble pulse, increasing as the disease advances. The mind is usually clear to the last, yet the little patient lies in that listless and indifferent condition, so common with typhoid patients, and dies from sheer exhaustion.

The duration of this disease is from six to sixteen days, running a most rapid course when commencing in the cheek or throat, but somewhat slower in its progress when commencing in the gums.

*Diagnosis.* This can be mistaken for no other disease. Its first stage is that of ulcerative stomatitis; but it differs from the simple form of this disease in the marked and constantly increasing debility of the patient. It also speedily assumes its diagnostic phagedenic character, becoming gangrenous usually in two or three days from its inception.

*Treatment.* This disease demands prompt and energetic treatment. As soon as an ulcer shows itself, I commence with the chlorate of potassa, in from five to ten grain doses every four to six hours, and continue the administration of this medicine—which is justly considered almost a specific in the simple form of stomatitis—during the whole progress of the disease. We must not, however, rely wholly upon this remedy. Our chief hope lies in preventing the ulcers from assuming a phagedenic character, and to meet this end, the part should be thoroughly cauterized as soon as any signs of sloughing appear. The child should be immediately transferred to a room separate from all other children, and every care taken that the disease be not communicated to others, and all cases of stomatitis then in the hospital should be at once cauterized, even before they assume the characteristic malignant features. Of all the caustics, I prefer the solid nitrate of silver, for most cases; yet it will often be necessary to use the acid nitrate of mercury, strong hydrochloric acid, bromine, or other powerful caustics; and even then we too often fail in our attempts to prevent the rapid spread of the disease. The mouth must be frequently washed with a weak solution of liquor sodæ chlorinatæ, oz.j to water oz.xij, and after mortification has commenced, a pledget of soft linen wet in this solution, should be constantly kept between the sore and the adjacent tissue. Tonics and stimulants should be freely given on account of the great prostration; and iron with bark or quinine in as heavy doses as the child will bear, and strong beef tea in place of solid food, which latter the child will almost invariably refuse to take, are the best.

*Prognosis.* Cancrum oris must be regarded as a peculiarly fatal form of stomatitis; in fact, every case that came under our observation, fourteen in number, when the disease was not checked before becoming gangrenous, died. Out of sixty-seven cases of stomatitis treated during the year, there were nineteen deaths, or twenty-eight per cent. This includes not only every case that was regarded as cancrum oris, but five others which, having the same characteristic features, proved fatal before the disease reached the gangrenous state.

The following report of cases, taken from my note book, may not be without interest:

*Case* 1. C. S., aged two and a half years, was admitted to the hospital, April 28th, with rubeola, having pneumonia as a complication. He recovered so far as to be up and playing in the convalescent ward, yet he did not gain strength.

June 10th. A small ulcer made its appearance at the junction of the frænum labii superioris with the gums, just between the two incisor teeth. I touched this ulcer with the solid nitrate of silver, and gave internally the chlorate of potassa in five grain doses every four hours, in addition to the tonic of quinine and iron that he had been taking for three weeks.

June 11th. The ulceration spreading. The two incisor teeth being very loose, I took them out of the way and again applied the nitrate of silver thoroughly to the ulcer.

June 12th. Upper lip much swelled; ulceration worse; applied bromine to the part. Washed the mouth frequently during the day and night with the following: R. Potass. Chlorat, dr.iv; Tr. Myrrhæ, oz.j; Tr. Ferri Chl., dr.iij; Syr. Aurant, oz.iv; Aquæ, oz.ij*ss*. *M.*

June 15th. Both cheeks swollen; upper lip protrudes; has lost another tooth. Bromine has been applied daily, and a cloth wet with liquor sodæ chlorinat. diluted with ten parts of water, kept constantly under the lip.

June 16th. The ulceration has eaten through the lip at the junction of the right ala of the nose, making a black gangrenous spot about the size of a dime. The lip is fully an inch thick.

June 18th. The gangrene extends about two inches in length, by an inch in width, including the end of the nose; the face is of a peculiar ashy-yellow color; both eyes are closed by the swelling; he is extremely weak.

June 21st. Died last evening. The mortification extends in a circle over three inches in diameter, destroying the whole nose, and including most of the upper lip. An autopsy revealed abundance of miliary tubercles throughout both lungs.

*Case* 2. J. T., aged two years, was admitted to the hospital May 22nd, with a light attack of rubeola. He gained no strength and did not wish to leave his bed. During the first week in June he passed by the mouth and nose, a number of worms—ascarides lumbricoides. He also had diarrhœa.

June 11th. His mouth is just beginning to be sore; a small ulcerated point is visible at the edge of the frænum labii superioris, at its junction with the gums; the gums are slightly inflamed and swollen. Touched the ulcer with nitrate of silver, and frequently during the day washed the mouth thoroughly with the following: R. Potass. Chlorat, dr.iv; Tr. Myrrhæ, oz.j; Tr. Ferri Chl., dr.iij; Syr. Aurantii, oz.iv; Aquæ, oz.ij*ss*. *M.* Gave internally chlorate of potassa in five grain doses, every four hours, in addition to the tonic of quinine and iron which he has been taking for some days.

June 12th. The ulceration is spreading along the upper jaw, and the inflammation has extended to the gums of the lower.

June 14th. Mouth worse. Changed the wash to: R. Cupri Sulph., dr.ij; Cinchonæ Pulv., oz.*ss*; Aquæ, oz.iv. *M.*, and apply with a brush.

June 15. This morning I noticed for the first time, on the left cheek, a small red spot, about the size of a pin's head, in the centre of a large red spot about the size of a quarter of a dollar. The cheek is much swollen, and on the inside, corresponding with the smaller spot, is an ulcer about the size of a dime, ash-colored, gangrenous, and emitting its peculiar offensive odor. Ordered the nurse to wash the mouth thoroughly with liquor sodæ chlor., diluted with four parts of water, and a cloth wet with this solution to be kept constantly between the jaw and the cheek.

June 16th. The small spot on the outside of the cheek has to-day the appearance of an ulcer. Cheek much swollen, hard and livid.

June 22nd. The swelling has gradually diminished. A black spot has this morning made its appearance on the left side of the nose, an inch and a half in length by half an inch in width, extending to the middle of the lip. It is of a bluish-black color, and slightly sunken from the rest of the face. Have poulticed the face to-day.

June 26th. Died this morning. The gangrenous portion has gradually increased in size, covering most of the left cheek and lip. No autopsy allowed.

*Case* 3. S. J. M., aged three years and nine months, entered the hospital, May 28th, with rubeola, complicated with pneumonia. Nothing unusual occurred till June 9th, when she threw up at least three ounces of dark clotted blood. She was much debilitated, had eaten no solid food since her admission, having lived on beef tea and milk punch. Ordered, Tinct. Kino.

June 10th. Noticed that her breath was slightly fœtid, and on examining the throat found it covered with a dirty ash-colored substance apparently membranous. Ordered, Potass. Chlorat. to be given in four grain doses, three times daily.

June 11th. A small ash-colored spot, about the size of a quarter of a dollar, has made its appearance on the roof of the mouth, near the molar teeth; the one in the throat is rapidly spreading. R. Potass. Chlorat., grs. v; Tr. Ferri Chl., *gtt.* v; *M.;* every three hours; also, wash the throat and mouth with: R. Cupri. Sulph., dr.ij; Cinchonæ Pulv., oz.*ss;* Aquæ, oz.iv. *M.*

June 13th. The throat and mouth are decidedly gangrenous, emitting a most offensive odor.

June 15th. She can swallow liquid only and with great difficulty. For the past two days she has apparently been wandering in mind, yet she answers all questions correctly.

June 16th. Died this morning, Autopsy revealed tubercles in both lungs, which had commenced to break down. There was some hepatization. Stomach and other organs normal. From the mouth to the sternum was a mass of gangrene.

*Case* 4. H. McL., aged two and a half years, entered the hospital July 2nd, with rubeola, which was soon complicated with bronchitis. She was closely watched for the first symptoms of stomatitis, as there were several cases of cancrum oris then under treatment, and as she was very weak. All went well till July 19th, when I noticed that her right cheek was hard, and swollen low down upon the submaxillary. An examination disclosed quite a gangrenous ulcer on the inside of the cheek. I immediately commenced with the following: R. Cupri Sulph., dr.ij; Cinchonæ Pulv., oz.*ss;* Aquæ, oz.iv; *M.;* brushing over the ulcerated surface thoroughly twice daily, and also inserting a cloth wet with liquor sodæ chlorinat, diluted with six parts of water, between the cheek and the jaw, renewing it every two or three hours. The child had been taking a tonic of iron and quinine for a week, and I now added to it chlorate of potassa, giving twenty grains daily in divided doses.

July 22nd. The swelling had greatly increased, and there was in the centre of the induration, a small black spot which rapidly increased in size, being at noon of the size of a half dime, and at night as large as a dime, somewhat sunken from the surrounding tissue. In the centre of this depression was a small opening into the cavity of the mouth, through which oozed, drop by drop, the offensive discharge from the gangrenous portion within.

July 23rd. The gangrenous part was fully an inch in diameter, and more sunken than before, the opening through it into the mouth being about a line in diameter; the face swelling greatly.

July 24th. The gangrene extended nearly to the corner of the mouth, being an inch and a half long and nearly as wide.

July 27th. The child died at five P. M., a most horrible object to behold. The gangrene had eaten out most of the cheek, extending from the symphysis to the angle of the jaw, and down on the neck, being over four inches in diameter. That portion of the lips and face not gangrenous, were very much swollen, closing both eyes, hard, and of a peculiar ashy-yellow color. There was no autopsy.

In the subjoined table, I have called the five cases where death occurred *prior* to the appearance of gangrene, malignant stomatitis, thereby placing none under the head of cancrum oris where gangrene was not a decidedly marked feature of the disease.

TABLE.

| No. of Case. | Sex. Male. | Sex. Female. | Age. Years. | Age. Months. | Death occurred in | Primary Disease. | Complications. | Sequelæ. | Autopsy. |
|---|---|---|---|---|---|---|---|---|---|
| 1 | 1 | -- | 2 | 6 | June | Rubeola | Pneumonia | Cancrum Oris | Miliar tubercles in both lungs. |
| 2 | -- | 1 | 2 | 6 | June | Marasmus | ---------- | Cancrum Oris | Mesentery filled with miliary tubercles. |
| 3 | 1 | -- | 2 | 7 | June | Rubeola | Pleuro-pneumonia | Cancrum Oris | Tubercles in both lungs ; vomicæ. |
| 4 | 1 | -- | 2 | -- | June | Rubeola | Diarrhœa ; ascarides | Cancrum Oris | |
| 5 | -- | 1 | 2 | -- | June | Rubeola | Pneumonia ; diarrhœa | Cancrum Oris | Tubercles in lungs, in abundance. |
| 6 | -- | 1 | 3 | 9 | June | Rubeola | Hematemesis ; pneumonia | Cancrum Oris | Tubercles in both lungs ; hepatization ; breaking down of the lung tissue. |
| 7 | -- | 1 | 4 | -- | July | Rubeola | Gastritis | Malignant Stomatitis | Tubercles in lungs ; congestion ; emphysema. |
| 8 | -- | 1 | 2 | 6 | July | Rubeola | Bronchitis | Cancrum Oris | |
| 9 | 1 | -- | 4 | 6 | August | Rubeola | Pneumonia ; laryngitis | Malignant Stomatitis | |
| 10 | 1 | -- | 4 | -- | Septembei | Rubeola | Conjunctivitis; Pustula Oph. | Cancrum Oris | |
| 11 | 1 | -- | 3 | -- | October | Rubeola | Tubercular Pneumonia | Cancrum Oris | Tubercles in lungs. |
| 12 | 1 | -- | 3 | -- | Octobei | Rubeola | Pneumonia | Cancrum Oris | |
| 13 | 1 | -- | 2 | 1 | Octobei | Rubeola | Pertussis ; Tracheitis | Cancrum Oris | Tubercles in lungs, with large vomica |
| 14 | 1 | -- | 2 | -- | November | Rubeola | Phthisis Pulmonalis | Malignant Stomatitis | Tubercles in lungs ; emphysema. |
| 15 | -- | 1 | 2 | -- | December | Rubeola | Prolapsus Ani | Cancrum Oris | Tubercles in lungs. |
| 16 | -- | 1 | 4 | -- | January | ---------- | Hæmoptisis ; pneumonia | Malignant Stomatitis | Tubercles in lungs ; had been in hospital for two years. |
| 17 | 1 | -- | 4 | 7 | February | Rubeola | Tubercular Pneumonia | Malignant Stomatitis | Tubercles in lungs. |
| 18 | 1 | -- | 1 | 8 | March | Rubeola | Otorrhœa | Cancrum Oris | Tubercles in lungs. |
| 19 | 1 | -- | 3 | -- | March | Phthisis Pul. | ---------- | Cancrum Oris | Tubercles in lungs. |

The table shows, that of the nineteen cases recorded, twelve were males and seven females. Their ages varied from one year and eight months, to four years and seven months, averaging two years and eleven months. Six deaths occurred during the month of June, two in July, one in August, one in September, three in October, one in November, one in December, one in January, one in February, and two in March. April and May were the only months of the year in which no death occurred from this disease. It should, however, be stated that two of the patients who died in June, were sick with disease during the last week of May, their deaths occurring on the 4th and 6th of June respectively.

In sixteen cases, the primary disease was rubeola. One entered the hospital having phthisis pulmonalis fully developed, one with marasmus, and one had been an inmate for over two years, having suffered from a variety of diseases, hæmoptisis and pneumonia immediately preceding the stomatitis.

It will be noted that in most of the cases, the complications were of a pulmonary character, and all of a debilitating nature. Fourteen autopsies were made; and in every case, tubercles were found in great abundance. In thirteen cases, the tubercles were found in the lungs, and in the other, the mesentery was filled with miliary tubercles.

Of the five cases in which there was no *post mortem* held, three are known to have had severe pulmonic complications, and all being much emaciated, we have reason to believe that these, too, would have exhibited tubercles, had they been examined.

These facts lead me to the conclusion, that cancrum oris has a close connection with tuberculosis; and, I believe, that it can exist only in children of a tuberculous diathesis.—*Western Lancet.*

